# Editorial: Herbal medicines in managing stroke and neurodegenerative diseases—Is there evidence based on basic and clinical studies?, volume II

**DOI:** 10.3389/fphar.2022.1059848

**Published:** 2022-11-03

**Authors:** Huazheng Liang, Haiyu Xu, Hui Zheng, Chunguang Li

**Affiliations:** ^1^ Translational Research Institute of Brain and Brain-Like Intelligence, Shanghai Fourth People’s Hospital, School of Medicine, Tongji University, Shanghai, China; ^2^ Institute of Chinese Materia Medica, China Academy of Chinese Medical Sciences, Beijing, China; ^3^ The Third Hospital/Acupuncture and Tuina School, Chengdu University of Traditional Chinese Medicine, Chengdu, China; ^4^ NICM Health Research Institute, Western Sydney University, Westmead, NSW, Australia

**Keywords:** herbal medicine, ethnopharmacology, plant derivatives, molecular mechanism, stroke, neurodegenerative disorders

Stroke is the leading cause of death in China and nearly 70% of it is ischemic stroke (Wang et al., 2017). Both thrombolysis and mechanical thrombectomy are recommended to treat ischemic stroke (Powers et al., 2019). However, the limited time windows or technical requirements for thrombolysis and mechanical thrombectomy render a large proportion of ischemic stroke patients ineligible for these treatments. Hemorrhagic stroke is more life threatening than ischemic stroke but it has no effective therapies available at the moment (Fernando et al., 2021). Neurodegenerative diseases are prevalent in the aging society, among which Alzheimer’s disease is the leading one and no effective therapy is available to cure this condition (Ismail et al., 2020). These challenges might be partially resolved by Traditional Chinese Medicine which has a long history and is known to effectively treat these conditions.

This Research Topic is a collection of 7 articles, including 2 original research articles, 2 study protocols, 2 systematic reviews, and 1 review.

In a systematic review of Li et al., the impact of natural platelet-activating factor receptor antagonists on acute ischemic stroke was analyzed. It was found that compounds derived from ginkgo like ginkgo endoterpene diester meglumine (GEDM), ginkgolide injection (GDI), ginkgo biloba dropping pill (GBDP) plus conventional medicine or edaravone or n-butylphthalide were better than conventional medicine alone in managing acute ischemic stroke. Medium and high doses of HSYA plus conventional medicine (50 and 70 mg/d, respectively) were more effective than Dengzhan Xixin injection, suggesting that herbs might be a source of potential therapeutics for ischemic stroke.

To overcome the limited time windows or technical requirements for thrombolysis and mechanical thrombectomy, Zhang et al. proposed a study to test the clinical efficacy of a herbal prescription - LongShengZhi capsule, a modified form of Buyang Huanwu decoction in a prospective, multi-center, randomized, placebo-controlled, double-blind, parallel-group, superiority trial. A total of 1,376 patients will be recruited and allocated to the intervention and control groups, respectively. Patients will take 2 g LongShengZhi capsules each time, three times a day, for 90 days. The primary outcome is the proportion of patients with favorable outcomes (mRS≤1). Secondary outcomes are distribution of mRS scores, proportion of patients with good functional status (mRS≤2), improvement in NIHSS, and other functional outcomes. In another study by Dong et al., a real-world study on Naoshuantong Capsules was designed to test the clinical efficacy of this herbal product on ischemic stroke patients whose duration of disease was primarily less than 14 days. 4,185 patients have been enrolled so far. The result of this study will inform clinicians of the effectiveness of this herbal treatment. Results of these two studies will demonstrate the therapeutic efficacy of these two herbal products.

Intracerebral hemorrhage (ICH) is less prevalent than ischemic stroke but is associated with higher mortality and morbidity (Fernando et al., 2021). Neuroinflammation peaks 3–7 days after ICH. One of the key molecules that regulate inflammation in the surrounding brain tissue and neuronal apoptosis is nuclear factor kappa B (NFκB) (Wagner, 2007). Mazhar et al. reported that herbal product-Zhilong Huoxue Tongyu capsules improved neurological functions through downregulating the expression of the total and phosphorylated NFκB-p65 and IKKβ, and a number of proinflammatory cytokines, such as TNFα, IL6, etc at both 24 and 72 h after starting the treatment, and upregulating the expression of total IKβα. Changes of TNFα and NFκB-p65 were observed not only in microglia, but also in neurons. These suggest that herbal products are optimal choices for ICH, at least in diminishing secondary injuries. In a study by Lin et al., meta-analysis was conducted on published clinical research on herbal recipes for ICH. It was found that combined use of these medicines with conventional western medicines were superior to conventional western medicines alone in improving the outcomes of ICH patients. *Conioselinum anthriscoides “Chuanxiong”* [Apiaceae], *Camellia reticulata Lindl.* [Theaceae], and *Bupleurum sibiricum var. jeholense (Nakai) C.D.Chu* [Apiaceae]) were the most frequently used herbs in the treatment of ICH. Diverse mechanisms were summarized, including anti-inflammatory and angiogenesis promoting effects. Therefore, herbs are also a source of therapeutics for increasing the absorption of the hematoma and alleviating edema.

In searching for alternative medicines for AD, Dong et al. reviewed the molecular mechanisms underlying the therapeutic effect of *Astragalus mongholicus* Bunge (Fabaceae) on AD as well as the potential bioactive compounds. It was found that this herb is used in a variety of herbal decoctions which are prescribed to treat AD, such as Buyang Huanwu decoction, etc. Over 20 compounds belonging to saponins, flavonoids, polysaccharides, and others were identified from the extract of *Astragalus mongholicus* Bunge (Fabaceae). Compounds purified from this herb are able to inhibit beta amyloid production, aggregation and tau hyperphosphorylation, protect neural cells from apoptosis, suppress neuroinflammation, and to promote neural stem cell proliferation and differentiation. Polysaccharides can even attenuate oxidative stress. Some of these compounds might become medications for AD patients in the future after validating their efficacy through clinical trials.

Perioperative neurocognitive disorders (PNDs) remain a challenge in the field of geriatric medicine. Neuroinflammation has been implicated in the pathogenesis of PNDs (Subramaniyan and Terrando, 2019). Chu et al. tested the therapeutic effect of the active fraction of *Sigesbeckia orientalis* L. on PNDs and purified the bioactive compounds. They found that the SO fraction from the D101 column showed the most prominent inhibitory effect on cultured microglia treated with LPS. In the laparotomy PND mouse model, significant cognitive impairment was observed, which was reversed by D101 fraction pretreatment. Inflammatory markers, including a variety of cytokines, and the intensity of Iba1- a marker for microglia, in the dentate gyrus of the hippocampus were significantly reduced by the D101 fraction pretreatment. So was the number of spines in the hippocampus. Active compounds, including rutin, isochlorogenic acid A, isochlorogenic acid C and darutoside, were identified from the D101 fraction. These suggest that compounds present in *Sigesbeckia orientalis* L. are able to restore cognitive performance of PND models partially through suppressing neuroinflammation.

It is our hope that the above studies will provide evidence on supporting the selection of alternative and complementary medicines to treat neurodegenerative diseases and stroke, at least will point to a new direction in our research on these conditions.

## References

[B1] Fernando S. M. Qureshi D. Talarico R. Tanuseputro P. Dowlatshahi D. Sood M. M. (2021). Intracerebral hemorrhage incidence, mortality, and association with oral anticoagulation use: A population study. Stroke 52 (5), 1673–1681. 10.1161/STROKEAHA.120.032550 33685222

[B2] Ismail Z. Black S. E. Camicioli R. Chertkow H. Herrmann N. Laforce R. Jr (2020). Recommendations of the 5th Canadian consensus conference on the diagnosis and treatment of dementia. Alzheimers Dement. 16 (8), 1182–1195. 10.1002/alz.12105 32725777 PMC7984031

[B3] Powers W. J. Rabinstein A. A. Ackerson T. Adeoye O. M. Bambakidis N. C. Becker K. (2019). Guidelines for the early management of patients with acute ischemic stroke: 2019 update to the 2018 guidelines for the early management of acute ischemic stroke: A guideline for healthcare professionals from the American heart association/American stroke association. Stroke 50 (12), e344–e418. 10.1161/STR.0000000000000211 31662037

[B4] Scheltens P. De Strooper B. Kivipelto M. Holstege H. Chételat G. Teunissen C. E. (2021). Alzheimer's disease. Lancet (London, Engl. 397 (10284), 1577–1590. 10.1016/S0140-6736(20)32205-4 PMC8354300 33667416

[B5] Subramaniyan S. Terrando N. (2019). Neuroinflammation and perioperative neurocognitive disorders. Anesth. Analg. 128 (4), 781–788. 10.1213/ANE.0000000000004053 30883423 PMC6437083

[B6] Wagner K. R. (2007). Modeling intracerebral hemorrhage: Glutamate, nuclear factor-kappa b signaling and cytokines. Stroke 38 (2), 753–758. 10.1161/01.STR.0000255033.02904.db 17261732

[B7] Wang W. Jiang B. Sun H. Ru X. Sun D. Wang L. NESS-China Investigators (2017). Prevalence, incidence, and mortality of stroke in China: Results from a nationwide population-based survey of 480 687 adults. Circulation 135 (8), 759–771. 10.1161/CIRCULATIONAHA.116.025250 28052979

